# Extracellular Vesicles in Regenerative Processes Associated with Muscle Injury Recovery of Professional Athletes Undergoing Sub Maximal Strength Rehabilitation

**DOI:** 10.3390/ijms232314913

**Published:** 2022-11-29

**Authors:** Giulia Catitti, Maria Concetta Cufaro, Domenico De Bellis, Ilaria Cicalini, Simone Vespa, Federico Tonelli, Giulia Miscia, Lorenzo Secondi, Pasquale Simeone, Vincenzo De Laurenzi, Damiana Pieragostino, Piero Del Boccio, Paola Lanuti

**Affiliations:** 1 Department of Medicine and Aging Sciences, University “G. d’Annunzio” of Chieti-Pescara, 66100 Chieti, Italy; 2 Center for Advanced Studies and Technology (CAST), University “G. d’Annunzio” of Chieti-Pescara, 66100 Chieti, Italy; 3 Department of Innovative Technologies in Medicine & Dentistry, University “G. d’Annunzio” of Chieti-Pescara, 66100 Chieti, Italy; 4 Plastic and Reconstructive Surgery, Department of Surgical Sciences, Tor Vergata University, 00133 Rome, Italy; 5 Department of Pharmacy, University “G. d’Annunzio” of Chieti-Pescara, 66100 Chieti, Italy

**Keywords:** platelet-derived extracellular vesicles, regenerative medicine, PRP, proteomics

## Abstract

Platelet-rich plasma (PRP) has great potential in regenerative medicine. In addition to the well-known regenerative potential of secreted growth factors, extracellular vesicles (EVs) are emerging as potential key players in the regulation of tissue repair. However, little is known about their therapeutic potential as regenerative agents. In this study, we have identified and subtyped circulating EVs (platelet-, endothelial-, and leukocyte-derived EVs) in the peripheral blood of athletes recovering from recent muscular injuries and undergoing a submaximal strength rehabilitation program. We found a significant increase in circulating platelet-derived EVs at the end of the rehabilitation program. Moreover, EVs from PRP samples were isolated by fluorescence-activated cell sorting and analyzed by label-free proteomics. The proteomic analysis of PRP-EVs revealed that 32% of the identified proteins were associated to “defense and immunity”, and altogether these proteins were involved in vesicle-mediated transport (GO: 0016192; FDR = 3.132 × 10^−19^), as well as in wound healing (GO: 0042060; FDR = 4.252 × 10^−13^) and in the events regulating such a process (GO: 0061041; FDR = 2.812 × 10^−12^). Altogether, these data suggest that platelet-derived EVs may significantly contribute to the regeneration potential of PRP preparations.

## 1. Introduction

Platelet-rich plasma (PRP) is a peripheral blood-derived preparation, containing higher concentrations of platelets than whole blood [1]. PRP has a great potential in regenerative medicine, given that its therapeutic properties were demonstrated in a wide range of clinical fields, including cardiac [2], maxillofacial [3], and plastic surgery, as well as orthopedics [4], dermatology [5], and sports medicine [6]. In addition to PRP’s well-known safety, convenience, and clinical potential, PRP’s mechanisms involved in regenerative medicine are not completely understood. Recently, it has been hypothesized that, in addition to secreted growth factors, extracellular vesicles (EVs) could have a role as key players in the PRP regulation of tissue repair [7,8,9]. EVs are cell-derived nanosized particles, surrounded by a lipid bilayer, and are similar, in terms of lipid composition (i.e., desatured lipids, sphingomyelinase and gangliosides), to that of cell plasma membranes [10]. EVs carry active cargoes, consisting of proteins, lipids, mRNAs, long/short noncoding RNAs, DNA fragments, and even organelles (i.e., mitochondria) [11]. Traditionally, three different EV subtypes, namely exosomes, microvesicles (MVs), and apoptotic bodies, were identified based on their biogenesis and size. Exosomes, the smallest EVs (with diameters ranging from 30 to 150 nm), originate within the lumens of multivesicular bodies (MVBs), and are released by exocytosis [12,13]. MVs generally range from 100 to 1000 nm in diameter and are released by blebbing or budding, therefore retaining the parental phenotype [14,15]. Apoptotic bodies, the larger EVs (~0.1 to ~5 µm), are released by apoptotic cells [16]. However, it has been underlined that the above reported classification does not fit the heterogeneity of the EV subtypes populations and their overlaps in size, cargoes, functions and biodistribution [17]. For this reason, the International Society of Extracellular Vesicles endorsed the use of the term “extracellular vesicle” for all EV subtypes, with a generic subclassification as small, if within 100 nm, and medium/large, if above 100–200 nm [18]. In any case, EVs have been identified in many body fluids, such as peripheral blood, cerebrospinal fluid, and tears [19,20,21,22,23,24,25]. EVs also express surface antigens that allow them to reach target cells [26]. Once attached the related recipient cell, EVs activate specific signaling responses interacting with their ligands; EVs can be also internalized by endocytosis and/or phagocytosis or can fuse with the membrane of the target cell, therefore delivering their cargo into the target cell cytosol, modifying the recipient cell biology [27]. In this case, EVs can directly activate the target cells by acting as signaling complexes, or can transfer genetic information, inducing transient or persistent phenotypic changes in recipient cells [28]. The involvement of EVs in the events regulating tissue, and in particular muscle, concerns the mechanisms of regeneration and repair [29]. The regeneration of damaged skeletal muscle largely relies on the presence of a population of cells, called satellite cells, that are mononuclear and myogenic elements that retain the capacity to proliferate and further differentiate to generate new fibers [30]. More recently, it has been also demonstrated that muscle tissue regeneration is a process characterized by a complex and coordinated interaction between muscles and the immune system [31]. In this context, it has been shown that after an acute injury, M1 cells infiltrate early during the first stages to promote the clearance of necrotic debris, whereas M2 macrophages develop later and sustain tissue healing [32]. More interestingly, as active messengers, leukocyte-derived, and more specifically macrophage-derived EVs have emerged as vital mediators in the mechanisms associated with tissue repair [33]. Recent data shows that platelet-derived EVs have also been involved in different healing responses [34].

Furthermore, even if a role for the EVs contained in PRP preparations (PRP-EVs) has been hypothesized [1], little is known about their therapeutic potential.

Here, we have analyzed leukocyte-, platelet- and endothelial-derived EV levels in a cohort of subjects recovering from muscle injuries. We showed that the concentrations of platelet-derived EVs was increased in the peripheral blood during the recovering phase. We have, therefore, purified PRP-EVs by fluorescence-activated cell sorting, to study their protein cargos with the final aim to understand their role as regenerative agents.

## 2. Results

### 2.1. Peripheral Blood EV Identification and Count in Athletes Recovering from Muscle Injuries

**Figure 1:**
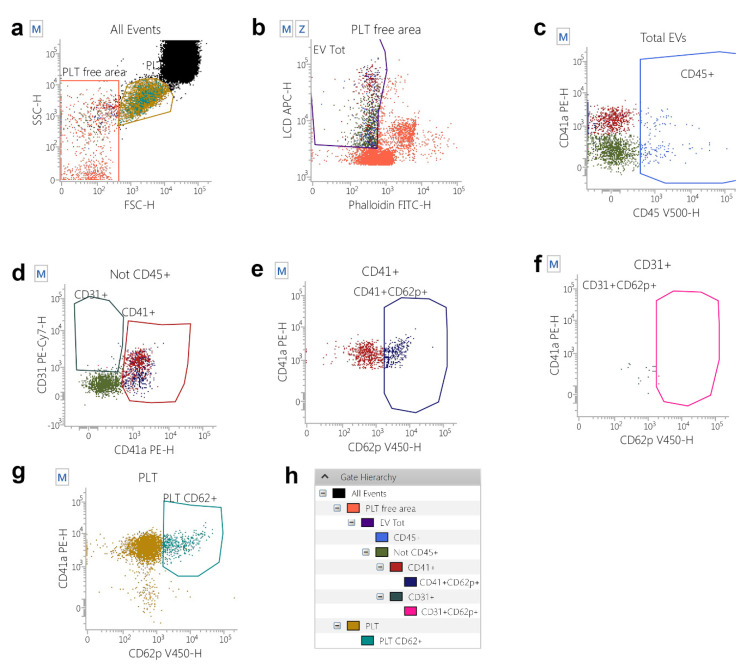
Gating Strategy for extracellular vesicles (EVs) identification and subtyping. (**a**) A platelet-free area region was defined on a Forward Scatter-H/Side Scatter-H dot-plot and, by using platelets (PLT) as a reference population. (**b**) The “Platelet-free area” was shown on a Phalloidin-H/Lipophilic Cationic Dye (LCD)-H dot-plot and EVs were identified as LCD positive/phalloidin negative events. (**c**) EVs (LCD+/Phalloidin- events) were analysed on a CD45-H/CD41a-H dot-plot and CD45+ events were identified as leukocyte-derived EVs. (**d**) A logical gate excluding all the CD45+ events was then obtained, and the resulting population was plotted on a CD31-H/CD41a-H dot-plot. Events showing the CD31+/CD41a+ phenotype were identified as platelet-derived EVs, whereas the CD31+/CD41a- compartment represented endothelium-derived EVs. (**e**) CD31+/CD41a+ platelet-derived EVs, CD31+/CD41a- endothelial-derived EVs (**f**) and whole platelets (**g**) were analysed for activation marker CD62P. (**h**) The applied gating hierarchy is shown as a scheme.

To study the possible physiological involvement of EVs in the processes associated with regeneration, a cohort of athletes recovering from muscle injuries (at T0) and undergoing submaximal strength rehabilitation was analyzed for the presence of EV of different phenotypes, as shown in the gating strategy depicted in Figure 1. As shown in Figure S1, no differences in terms of EV counts between athletes recovering from muscle injuries (at T0) and control subjects were evidenced.

**Figure 2:**
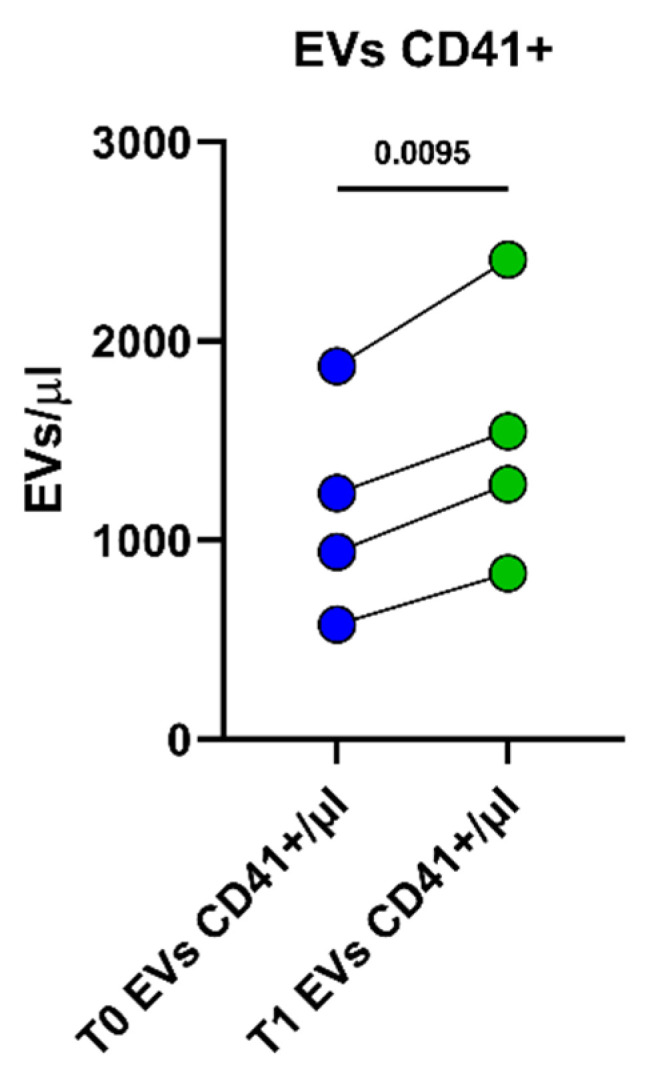
CD41+ circulating EVs in the peripheral blood of athletes recovering from muscle injuries. The graph shows absolute counts a of CD41+ circulating EVs, analyzed before (T0, blue dots) and after (T1, green dots) the rehabilitation program.

More interestingly, when athletes were measured before starting the rehabilitation (T0) and at the end of the program (T1), a significant increase of platelet-derived EVs was observed (*p* = 0.0095, Figure 2), whereas the other analyzed EV phenotypes did not change in terms of concentrations (Figure S2).

### 2.2. Phenotypes of PRP-EVs

**Figure 3:**
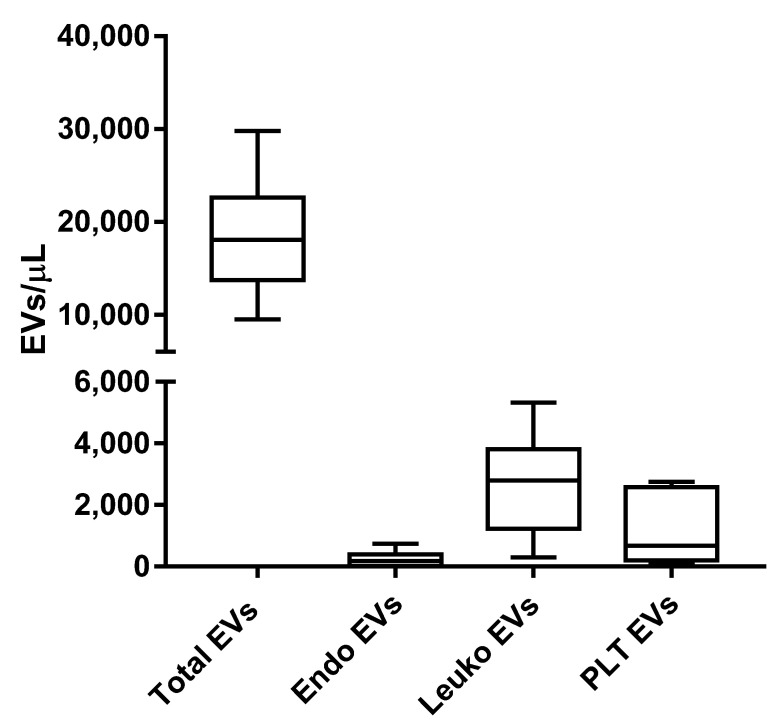
EV subtypes from PRP samples. The whole EV compartment, as well as EVs derived from the endothelium, leukocytes, and platelets were analyzed from the PRP of six subjects.

The EVs from six PRP samples, isolated by fluorescence-activated cell sorting, were subtyped using the panel described in Table S1 and the gating strategy depicted in Figure 1. Figure 3 shows the Box and Whiskers Plots, representing the distribution of the EV subtypes that we have analyzed. Leukocyte-derived EVs (Leuko EVs) are the most represented (mean = 2677.27 + 1706.11) EV subpopulation, followed by platelet-derived EVs (PLT EVs, mean = 1163.4 + 1193.52) and EVs stemming from the endothelium (Endo EVs, mean = 232.4 + 269.70).

### 2.3. Proteomic Characterization of the PRP-EV Cargo

1 × 10^6^ PRP-EVs isolated by fluorescence-activated cell sorting (FACS) were analyzed by shotgun proteomics from four different patients. Table 1 shows the list of the quantified proteins common in at least two of the analyzed samples.

**Figure 4:**
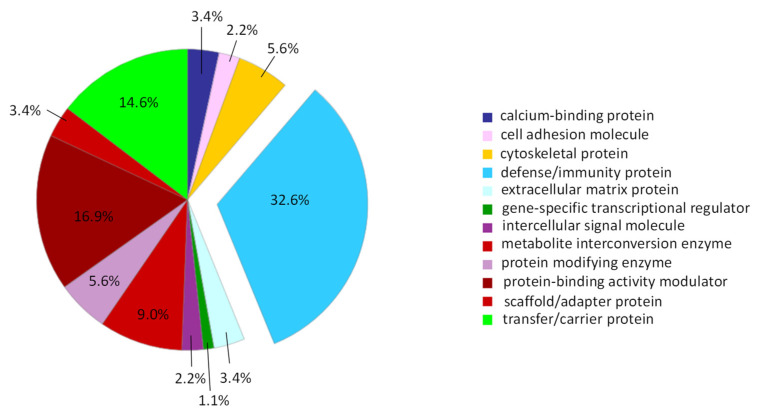
Protein classification viewed in pie charts of the 105 PRP-EV proteins reported in Table 1.

**Figure 5:**
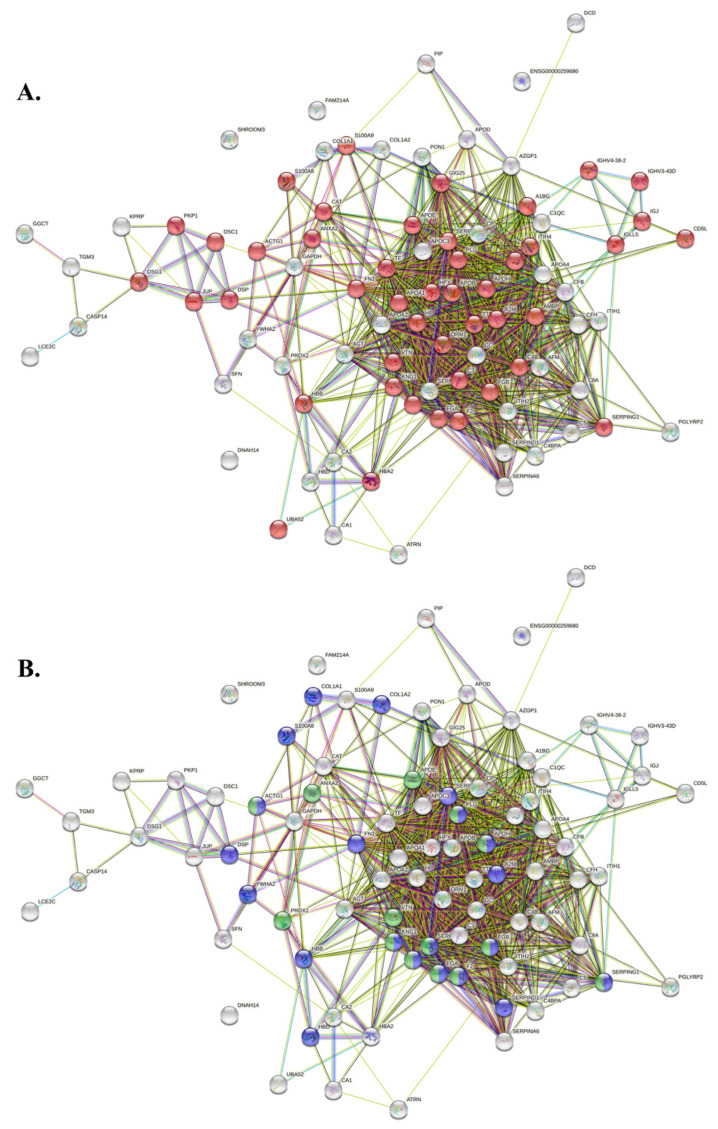
Network representing the interactions existing among the 105 PRP-EV proteins reported in Table 1. (**A**) Red dots represent the proteins involved in the “vesicle-mediated transport”. (**B**) Blue dots represent the proteins associated with “wound healing”, whereas green dots are related to the proteins regulating the wound healing process.

Panther protein classification analysis was performed and uploading PRP-EV proteins are listed in Table 1 with proteins grouped according to their biological functions. As shown in Figure 4, more than 32% of PRP-EV proteins are involved in the “defense and immunity” biological function. The String analysis (PPI enrichment *p*-value < 10^−16^) of PRP-EV proteins listed in Table 1 was carried out and the results, shown in Figure 5, demonstrated that they were mainly involved in vesicle-mediated transport (GO: 0016192; FDR = 3.132 × 10^−19^) (Figure 5A), as well as in wound healing (GO: 0042060; FDR = 4.252 × 10^−13^) and in the events regulating such a process (GO: 0061041; FDR = 2.812 × 10^−12^, Figure 5B).

## 3. Discussion

Platelet-derived EVs represent most of the peripheral blood circulating EVs, and, for this reason, they have a long history of discovery [35,36]. Traditionally, platelet-derived EVs have been described as procoagulant agents [37,38]. On the other hand, it is well known that PRP has great potential in promoting tissue repair and regeneration [39,40]. PRP is enriched in platelets and contains extracellular vesicles (EVs) [41], which have also attracted great interest in regenerative medicine [7,8,9].

**Figure 6:**
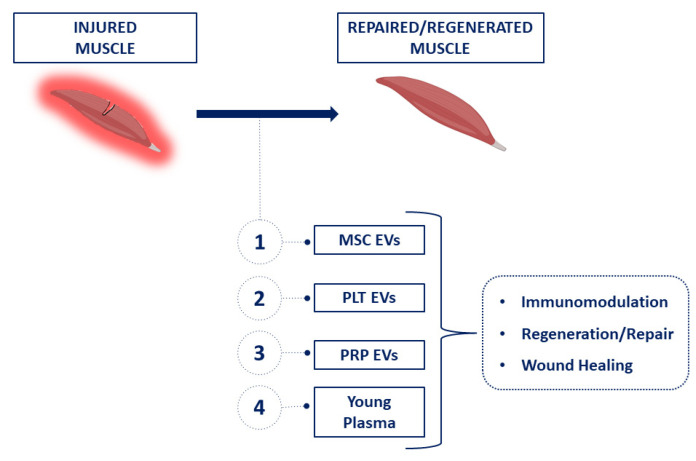
Scheme of EV interaction in the muscle repair process.

It is known that an injury-responsive production of EVs exists, which carry specific proteins, lipids, RNAs, and DNA fragments that facilitate tissue repair and regeneration. This EV cargo appears to be selectively packaged, depending on the context, the type of injury, and the cellular targets. Therefore, EVs produced upon muscle injuries participate in the orchestration of responses from myofiber repair and regeneration [29]. In detail, EVs from young serum play a key role in the rejuvenating effects exerted on aged skeletal muscles [42]. Several other studies demonstrated that mesenchymal-derived EVs (MSC-EVs) promoted myogenesis and angiogenesis in vitro, and muscle regeneration in in vivo models of muscle injury [43]. Such a regeneration process may be mediated, at least in part by miRNAs (i.e., *mir-494*) [44]. In this context, EVs are involved in a complex intercellular crosstalk process, and MSC-EVs, circulating in the body fluids, passing across the biological barriers, home to damaged tissues, participate in the repair of injured skeletal muscles [45]. Furthermore, adipose stem cell-derived EVs display skeletal muscle protective properties, associated with their cargo, which is enriched in *Neuregulin-1*/mRNA [46]. It has been also demonstrated that platelet-derived EVs carry many growth factors, such as VEGF, bFGF, TGF-β1, and PDGF-BB, as well as cytokines [7,47]. More specifically, it has been shown that PRP-EVs induce immunomodulatory effects and accelerate muscle recovery after injury in rat models [47,48]. A summary of the most important EVs in muscle regeneration is reported in Figure 6.

Therefore, we have analyzed the concentrations of EVs in the peripheral blood samples of professional athletes recovering from muscle injuries. Platelet-, leukocyte- and endothelial-derived EVs, already reported to be released during exercise [49], were characterized in subjects undergoing a recovering program including submaximal strength rehabilitation after the completion of such a program. Our data show that, during the recovery, the compartment of platelet-derived EVs significantly increased, suggesting that platelet-derived EVs may be involved in the processes driving muscle repair and regeneration. It can therefore be hypothesized that platelet-derived EVs may have a role in the muscle repair/regeneration during this phase, possibly also exerting anti-inflammatory effects by reshaping the inflammatory environment, as it was also demonstrated for rheumatoid arthritis-affected mice [50].

We have therefore carried out, for the first time to our knowledge, an in-depth analysis of the PRP-EV phenotypes and cargoes. We observed that the PRP-EVs have heterogeneous phenotypes, including leukocyte- and endothelial-derived EVs, even if platelet-derived EVs are well represented, as it was already observed [51].

To deeply study the protein cargo of PRP EVs, we have carried out a shotgun proteomics experiment. From the related molecular and functional characterization, 105 proteins were identified, mostly classified in the “defense and immunity” biological function. This data underlines that PRP EVs actively participate in the well-known interactions between muscle and immune system, orchestrating skeletal muscle regeneration [31]. Unfortunately, there are not accepted specific markers for the immune EV subtyping [18], but we can hypothesize that the leukocyte-derived EV compartment circulating in the peripheral blood of athletes affected by muscle injuries and therefore present in PRP preparations stems from the wide variety of innate and adaptive immune cells that have been involved in muscle repair processes [52]. These data are in line with previously reported evidence demonstrating that EVs convey immunomodulatory messengers that have been already involved in tissue repair and regeneration. Interestingly, PRP EVs that we have isolated by fluorescence-activated cell sorting showed a protein cargo associated with the vesicle-mediated transport. We observed that 49 out of 105 proteins were implicated in the “vesicle-mediated transport” function, demonstrating how the here-applied integrated EV sort-omics approach brings out typical proteins of EVs, potentially implicated in conveying biological information [22,25].

Therefore, those EVs may mediate the intercellular crosstalk, passing across the biological barriers, and reaching remote target cells and tissues [11]. Indeed, our data demonstrate that the cargo of PRP EVs plays a main role in conveying information related to regeneration processes. Furthermore, among the functional networks, “wound healing” emerged as a significant biological function contextually related to “vesicular-mediated transport”, given that more than 80% of wound healing-related proteins were also associated to the vesicle-mediated transport function.

Therefore, PRP EVs may have great potential in promoting tissue repair and regeneration. Some disadvantages have been associated with the use of whole PRP-based therapies. It has been demonstrated that many PRP-derived active biomolecules are damaged by lytic enzymes from the extracellular milieu, not being protected by the plasma membranes, and losing their activities [1]. Additionally, even if platelets lack an integral cell structure, cases of immunological rejection between allogeneic individuals have been registered [1]. On the other hand, several advantages have been demonstrated to be associated with the use of EVs, including their scarce immunogenicity and their ability to be locally released [1]. Interestingly, the use of purified EVs instead of whole PRP preparations as regenerative agents may, therefore, circumvent all these limitations.

Here, we combined, for the first time to our knowledge, the analysis of circulating EVs in untouched peripheral blood samples of athletes recovering from recent muscular injuries with the analysis of the cargo of PRP preparations. The major limitation of this study is the small number of enrolled subjects, even if both flow cytometry and proteomics data underline a possible relevant role of platelet-derived EVs in the processes regulating both repair and regeneration of muscle tissue. In this context, EV-based subcellular therapies have great potential for clinical applications in regenerative medicine, overcoming the challenges of cell-based therapies. Therefore, even if further enlarged studies are needed, our data offer exciting new insights into the complex process of muscle repair, underlying that the use of EVs can be optimized for therapeutic purposes in disease as well as in sport.

## 4. Materials and Methods

### 4.1. Patients

The study, carried out according to the ethical principles laid down by the latest version of the Declaration of Helsinki, was approved by the Ethical Committee of the University “G. d’Annunzio”, Chieti-Pescara, Italy (approval code: 29012020). Written informed consent was obtained from all study participants. All patients were enrolled from January to November 2021. We enrolled 4 athletes recovering from recent muscular injuries (all males, mean age: 25.2 + 3.8) and 3 healthy volunteers who did not reported any injury during the last 5 years, and matched them for age and sex with recently injured athletes. We also enrolled 6 athletes recovering from injuries whose peripheral blood samples were used to produce PRP samples. To identify, count, and subtype circulating EVs, peripheral blood samples were collected from all control subjects and from recently injured athletes before (T0) starting a recovering program including submaximal strength rehabilitation and strength development and after (T1) the rehabilitation program [53,54].

### 4.2. Flow Cytometry Identification, Count, and Subtyping of EVs from Peripheral Blood Samples

Peripheral blood samples were drawn using sodium citrate tubes (Becton Dickinson Biosciences-BD, San Jose, CA, USA, Ref 454387) and analyzed within 4 h from venipuncture. The EV staining was carried out as previously described [21,55,56,57]. Briefly, the reagent mix summarized in Table S1 was added to 195 μL of PBS 1X; then, 5 μL of whole blood were added to the mix. After 45 min of staining (RT, in the dark), 500 μL of PBS 1X were added to each tube and 1 × 10^6^ events/sample were acquired by flow cytometry (FACSVerse, BD Biosciences, San Jose, CA, USA), placing the trigger threshold on the lipophilic cationic dye channel (LCD) [58]. To avoid immune complex formation and antibody aggregation, each reagent stock solution was centrifuged before its use (21,000× *g*, 12 min). For all measured parameters, the height (H) signals are shown.

Instrument performances, data reproducibility, and fluorescence calibrations were monitored by the Cytometer Setup and Tracking Module (BD Biosciences). The evaluation of non-specific fluorescence was obtained by acquiring FMO combined with the respective isotype control [59,60]. Data were analyzed using FACSuite v 1.0.6.5230 (BD Biosciences) and FlowJo X v 10.0.7 (BD Biosciences) software. EVs concentrations were calculated based on the volumetric count function.

### 4.3. Flow Cytometry Gating Strategy

Figure 1A shows the gating strategy used to identify, count, and subtype circulating EVs. In detail, a forward scatter height (FSC-H)/ Side Scatter-H (SSC-H) dot-plot was used to establish a region, defined as “platelet free area”, containing the events with the EV scatter features (Figure 1A) [55,56]. Those events were represented on an LCD-H/Phalloidin-H dot-plot and EVs were identified as LCD positive/phalloidin negative dots (Figure 1B). Therefore, EVs (LCD+/Phalloidin- events) were analyzed on a CD45-H/CD41a-H dot-plot and CD45+ events were identified as leukocyte-derived EVs (Figure 1C) [18]. A CD45 negative logical gate was obtained, and the resulting population was represented on a CD31-H/CD41a-H dot-plot. Events showing the CD31+/CD41a+ phenotype were identified as platelet-derived EVs (Platelet EVs) (Figure 1D) [18], whereas the compartment was identified as endothelial-derived EVs (Figure 1D) [18]. Platelet-derived EVs (Figure 1E) and endothelial-derived EVs (Figure 1F) and all platelets (Figure 1G) were further analyzed for the activation marker CD62P. In Figure 1H, the used gating hierarchy is shown as a scheme.

### 4.4. Platelet-Enriched Plasma Preparation

The preparation of PRP was carried out as already reported [61]. Briefly, peripheral blood samples (60 mL) were drawn in 10% citrate dextrose A (ACD) solution and centrifuged (600× *g*, 10 min). The plasma fraction was then centrifuged at 4000× *g* for 15 min. Supernatants, representing the platelet poor plasma (PPP), were removed, and the remaining suspension, the PRP, was collected and mixed with the platelets present in the PPP, previously trapped by filtration. The mixture was passed through a WBC filter (Terumo Imuguard, CO, USA) to remove the leukocyte fraction. Platelet and leukocyte counts were carried out, and platelet concentrations were adjusted to 1 × 10^10^ in 8 mL.

### 4.5. EV Separation by Fluorescence-Activated Cell Sorting

PRP-EVs were isolated by fluorescence activated cell sorting, as previously reported [55]. Briefly, PRP samples were stained by 0.5 µL of FITC-conjugated phalloidin and LCD (BD Biosciences–Catalog, #626267, Custom Kit), as described above. After 45 min of staining (RT, in the dark), at least 500 µL of PBS 1X was added to each tube. Such a dilution allowed us to maintain the correct event rate recommended for 100 μm nozzle, which we have used. Total EVs (LCD+/Phalloidin- events) were separated using a FACSAria III cell sorter (BD Biosciences San Jose, CA, USA). The trigger threshold was placed on the APC channel and, for all parameters, the height (H) signals, as well as bi-exponential or logarithmic modes were selected. The post-sorting purity was assessed by using the same instrument (FACSAria III) and the same setting applied for EV separation. Instrument performances, data reproducibility, and fluorescence calibrations were sustained by the Cytometer Setup and Tracking Module (BD Biosciences).

### 4.6. EV Protein Cargo Detection by Label-Free Proteomics

Proteomics analyses were normalized by using the number of purified EVs. As already described, 1 × 10^6^ purified PRP-EVs were used for each proteomic detection [22,25]. Digested proteins were acquired in triplicate by LC-MS/MS using the UltiMate^TM^ 3000 UPLC (Thermo Fisher Scientific, Waltham, MA, USA) chromatographic system coupled to the Orbitrap Fusion^TM^ Tribrid^TM^ (Thermo Fisher Scientific, Waltham, MA, USA) mass spectrometer. Briefly, the flow rate was set at 300 nL/min with a total run of 65 min by using an EASY-spray Acclaim^TM^ PepMap^TM^ C18 (75 μm ID, 25 cm L, 2 μm PS, Thermo Fisher Scientific) nanoscale chromatographic column. Details of LC-MS/MS parameters are reported in our previous work [62]. Proteomics MS/MS raw data were processed using the Andromeda peptide search engine through MaxQuant version 1.6.10.50 (Max-Planck Institute for Biochemistry, Martinsried, Germany) matching spectra against the UniProt database (released 2020_06, taxonomy Homo Sapiens, 20,588 entries) supplemented with frequently observed contaminants and containing forward and reverse sequences. iBAQ (intensity-based absolute quantification) values were used to quantify protein abundance in each sample whenever the protein was quantified in at least two analytical replicates. STRING version 11.5 database was used to perform Protein–Protein Interaction (PPI) networks.

### 4.7. Statistical Analysis

Data were analyzed using the XLSTAT 2022 (Addinsoft, Paris, France) and GraphPad Prism 9 (GraphPad Software Inc., La Jolla, CA, USA). Two-sided Student’s *t*-test or paired *t*-test were used as indicated. Statistical significance was accepted for *p* < 0.05.

## Figures and Tables

**Table 1:** The represented list has been obtained by identifying the proteins displayed by at least two PRP-EV isolated samples. The Uniprot code and the Gene name, Protein full name, Protein Classification, and Functional Network interaction are reported. WH means Wound Healing, VH means Vesicle-mediated Transport.

Protein UniProt Code	Gene Name	Protein Name	Protein Class	Functional Network Interaction
P01834	IGKC	Immunoglobulin kappa constant	defense/immunity protein	-
P01860	IGHG3	Immunoglobulin heavy constant gamma 3	defense/immunity protein	-
A0A0C4DH42	IGHV3–66	Immunoglobulin heavy variable 3–66	defense/immunity protein	-
P0DOY3	IGLC3	Immunoglobulin lambda constant 3	defense/immunity protein	-
P0DP04	IGHV3–43D	Immunoglobulin heavy variable 3–43D	defense/immunity protein	VT
P01857	IGHG1	Immunoglobulin heavy constant gamma 1	defense/immunity protein	-
P02749	APOH	Beta-2-glycoprotein 1	defense/immunity protein	VT
P01876	IGHA1	Immunoglobulin heavy constant alpha 1	defense/immunity protein	-
P0DP01	IGHV1–8	Immunoglobulin heavy variable 1–8	defense/immunity protein	-
P25311	AZGP1	Zinc-alpha-2-glycoprotein	defense/immunity protein	-
A0A075B7B8	IGHV3OR16–12	Immunoglobulin heavy variable 3/OR16–12 (non-functional)	defense/immunity protein	-
P01871	IGHM	Immunoglobulin heavy constant mu	defense/immunity protein	-
A0A0C4DH31	HV1–18	Immunoglobulin heavy variable 1–18	defense/immunity protein	-
P08603	CFH	Complement factor H	defense/immunity protein	-
P0DP08	IGHV4–38–2	Immunoglobulin heavy variable 4–38–2	defense/immunity protein	VT
P0DP03	HV3–30–5	Immunoglobulin heavy variable 3–30–5	defense/immunity protein	-
P01591	IGJ	Immunoglobulin J chain	defense/immunity protein	VT
P04003	C4BPA	C4b-binding protein alpha chain	defense/immunity protein	VT
A2NJV5	KV2–29	Immunoglobulin kappa variable 2–29	defense/immunity protein	-
P0CG04	IGLL5	Immunoglobulin lambda like polypeptide 5	defense/immunity protein	VT
P01861	IGHG4	Immunoglobulin heavy constant gamma 4	defense/immunity protein	-
P01619	KV320	Immunoglobulin kappa variable 3–20	defense/immunity protein	-
A0A0B4J1V6	HV373	Immunoglobulin heavy variable 3–73	defense/immunity protein	-
P07357	C8A	Complement component C8 alpha chain	defense/immunity protein	-
Q96PD5	PGLYRP2	N-acetylmuramoyl-L-alanine amidase	defense/immunity protein	-
P01877	IGHA2	Immunoglobulin heavy constant alpha 2	defense/immunity protein	-
P01859	IGHG2	Immunoglobulin heavy constant gamma 2	defense/immunity protein	-
Q0VDD8	DNAH14	Dynein axonemal heavy chain 14	cytoskeletal protein	-
Q8TF72	SHROOM3	Protein Shroom3	cytoskeletal protein	-
Q13835	PKP1	Plakophilin-1	cytoskeletal protein	VT
P15924	DSP	Desmoplakin	cytoskeletal protein	WH, VT
P63261	ACTG1	Actin, cytoplasmic 2	cytoskeletal protein	WH, VT
P69905	HBA2	Hemoglobin subunit alpha 2	transfer/carrier protein	VT
P68871	HBB	Hemoglobin subunit beta	transfer/carrier protein	WH, VT
P02787	TF	Serotransferrin	transfer/carrier protein	VT
P02042	HBD	Hemoglobin subunit delta	transfer/carrier protein	WH
P02656	APOC3	Apolipoprotein C-III	transfer/carrier protein	-
P02774	GC	Vitamin D-binding protein	transfer/carrier protein	-
P04114	APOB	Apolipoprotein B-100	transfer/carrier protein	VT
P05090	APOD	Apolipoprotein D	transfer/carrier protein	-
P43652	AFM	Afamin	transfer/carrier protein	-
P06727	APOA4	Apolipoprotein A-IV	transfer/carrier protein	-
P02649	APOE	Apolipoprotein E	transfer/carrier protein	WH, VT
P02652	APOA2	Apolipoprotein A-II	transfer/carrier protein	-
P02647	APOA1	Apolipoprotein A-I	transfer/carrier protein	VT
P00738	HP	Haptoglobin	protein-modifying enzyme	VT
P00734	F2	Prothrombin	protein-modifying enzyme	WH, VT
P00747	PLG	Plasminogen	protein-modifying enzyme	WH, VT
P31944	CASP14	Caspase-14	protein-modifying enzyme	-
P02790	HPX	Hemopexin	protein-modifying enzyme	VT
P02675	FGB	Fibrinogen beta chain	intercellular signal molecules	WH, VT
P02679	FGG	Fibrinogen gamma chain	intercellular signal molecules	WH, VT
P01024	C3	Complement C3	protein-binding activity modulator	VT
P01009	SERPINA1	Alpha-1-antitrypsin	protein-binding activity modulator	WH, VT
P19827	ITIH1	Inter-alpha-trypsin inhibitor heavy chain H1	protein-binding activity modulator	-
P01011	GIG25	Serpin peptidase inhibitor, clade A (alpha-1 antiproteinase, antitrypsin), member 3	protein-binding activity modulator	VT
P01042	KNG1	Kininogen-1	protein-binding activity modulator	WH, VT
P01008	SERPINC1	Antithrombin-III	protein-binding activity modulator	WH
P19823	ITIH2	Inter-alpha-trypsin inhibitor heavy chain H2	protein-binding activity modulator	-
P01019	AGT	Angiotensinogen	protein-binding activity modulator	-
P05155	SERPING1	Plasma protease C1 inhibitor	protein-binding activity modulator	WH, VT
P0C0L5	C4B	Complement C4-B	protein-binding activity modulator	-
P01031	C5	Complement C5	protein-binding activity modulator	-
Q14624	ITIH4	Inter-alpha-trypsin inhibitor heavy chain H4	protein-binding activity modulator	VT
P05546	SERPIND1	Heparin cofactor 2	protein-binding activity modulator	WH
P08185	SERPINA6	Corticosteroid-binding globulin	protein-binding activity modulator	-
P01023	A2M	Alpha-2-macroglobulin	protein-binding activity modulator	WH, VT
P02452	COL1A1	Collagen alpha-1(I) chain	extracellular matrix protein	WH, VT
P02751	FN1	Fibronectin type III domain containing	extracellular matrix protein	VT
P08123	COL1A2	Collagen alpha-2(I) chain	extracellular matrix protein	WH, VT
P06702	S100A9	Protein S100-A9	calcium-binding protein	VT
P07355	ANXA2	Annexin A2	calcium-binding protein	WH, VT
P05109	S100A8	Protein S100-A8	calcium-binding protein	WH, VT
P00915	CA1	Carbonic anhydrase 1	metabolite interconversion enzyme	-
P00918	CA2	Carbonic anhydrase 2	metabolite interconversion enzyme	-
P02766	TTR	Transthyretin	metabolite interconversion enzyme	VT
P32119	PRDX2	Peroxiredoxin-2	metabolite interconversion enzyme	WH
P04406	GAPDH	Glyceraldehyde-3-phosphate dehydrogenase	metabolite interconversion enzyme	-
P04040	CAT	Catalase	metabolite interconversion enzyme	VT
Q08188	TGM3	Protein-glutamine gamma-glutamyltransferase E	metabolite interconversion enzyme	-
P00450	CP	Ceruloplasmin	metabolite interconversion enzyme	-
P02747	C1QC	Complement C1q subcomponent subunit C	scaffold/adaptor protein	-
P31947	SFN	14–3–3 protein sigma	scaffold/adaptor protein	VT
P63104	YWHAZ	14–3–3 protein zeta/delta	scaffold/adaptor protein	WH, VT
O75882	ATRN	Attractin	gene-specific transcriptional regulator	-
Q08554	DSC1	Desmocollin-1	cell adhesion molecule	VT
Q02413	DSG1	Desmoglein-1	cell adhesion molecule	VT
P12273	PIP	Prolactin induced protein	-	-
P02763	ORM1	Alpha-1-acid glycoprotein 1	-	VT
P02671	FGA	Fibrinogen alpha chain	-	WH, VT
S4R460	ENSG00000259680	Uncharacterized protein	-	-
P81605	DCD	Dermcidin	-	-
P10909	CLU	Clusterin	-	VT
P02760	AMBP	Protein AMBP	-	VT
P04217	A1BG	Alpha-1B-glycoprotein	-	VT
P27169	PON1	Serum paraoxonase/arylesterase 1	-	-
O43866	CD5L	CD5 antigen-like	-	VT
P04004	VTN	Vitronectin	-	WH, VT
P62987	UBA52	Ubiquitin-60S ribosomal protein L40	-	VT
P00751	CFB	Complement factor B	-	-
O75223	GGCT	Gamma-glutamylcyclotransferase	-	-
P14923	JUP	Junction plakoglobin	-	VT
Q32MH5	FAM214A	Protein FAM214A	-	-
Q5T749	KPRP	Keratinocyte proline rich protein	-	-
Q5TA81	LCE2C	Late cornified envelope protein 2C	-	-

## Data Availability

Data will be available under reasonable requests.

## Supplementary Materials

The following supporting information can be downloaded at: https://www.mdpi.com/article/10.3390/ijms232314913/s1.

## References

[1] Wu J., Piao Y., Liu Q., Yang X. (2021). Platelet-rich plasma-derived extracellular vesicles: A superior alternative in regenerative medicine?. Cell Prolif..

[2] Patel A.N., Selzman C.H., Kumpati G.S., McKellar S.H., Bull D.A. (2016). Evaluation of autologous platelet rich plasma for cardiac surgery: Outcome analysis of 2000 patients. J. Cardiothorac. Surg..

[3] Anitua E., Fernández-de-Retana S., Alkhraisat M.H. (2021). Platelet rich plasma in oral and maxillofacial surgery from the perspective of composition. Platelets.

[4] Muchedzi T.A., Roberts S.B. (2018). A systematic review of the effects of platelet rich plasma on outcomes for patients with knee osteoarthritis and following total knee arthroplasty. Surgeon.

[5] Emer J. (2019). Platelet-Rich Plasma (PRP): Current Applications in Dermatology. Skin Ther. Lett..

[6] Dohan Ehrenfest D.M., Andia I., Zumstein M.A., Zhang C.-Q., Pinto N.R., Bielecki T. (2014). Classification of platelet concentrates (Platelet-Rich Plasma-PRP, Platelet-Rich Fibrin-PRF) for topical and infiltrative use in orthopedic and sports medicine: Current consensus, clinical implications and perspectives. Muscles. Ligaments Tendons J..

[7] Torreggiani E., Perut F., Roncuzzi L., Zini N., Baglìo S.R., Baldini N. (2014). Exosomes: Novel effectors of human platelet lysate activity. Eur. Cells Mater..

[8] Tao S.-C., Yuan T., Rui B.-Y., Zhu Z.-Z., Guo S.-C., Zhang C.-Q. (2017). Exosomes derived from human platelet-rich plasma prevent apoptosis induced by glucocorticoid-associated endoplasmic reticulum stress in rat osteonecrosis of the femoral head via the Akt/Bad/Bcl-2 signal pathway. Theranostics.

[9] Guo S.-C., Tao S.-C., Yin W.-J., Qi X., Yuan T., Zhang C.-Q. (2017). Exosomes derived from platelet-rich plasma promote the re-epithelization of chronic cutaneous wounds via activation of YAP in a diabetic rat model. Theranostics.

[10] Zaborowski M.P., Balaj L., Breakefield X.O., Lai C.P. (2015). Extracellular Vesicles: Composition, Biological Relevance, and Methods of Study. Bioscience.

[11] Simeone P., Bologna G., Lanuti P., Pierdomenico L., Guagnano M.T., Pieragostino D., Del Boccio P., Vergara D., Marchisio M., Miscia S. (2020). Extracellular Vesicles as Signaling Mediators and Disease Biomarkers across Biological Barriers. Int. J. Mol. Sci..

[12] György B., Szabó T.G., Pásztói M., Pál Z., Misják P., Aradi B., László V., Pállinger E., Pap E., Kittel A. (2011). Membrane vesicles, current state-of-the-art: Emerging role of extracellular vesicles. Cell. Mol. Life Sci..

[13] Colombo M., Raposo G., Théry C. (2014). Biogenesis, secretion, and intercellular interactions of exosomes and other extracellular vesicles. Annu. Rev. Cell Dev. Biol..

[14] Cufaro M.C., Pieragostino D., Lanuti P., Rossi C., Cicalini I., Federici L., De Laurenzi V., Del Boccio P. (2019). Extracellular Vesicles and Their Potential Use in Monitoring Cancer Progression and Therapy: The Contribution of Proteomics. J. Oncol..

[15] Akers J.C., Gonda D., Kim R., Carter B.S., Chen C.C. (2013). Biogenesis of extracellular vesicles (EV): Exosomes, microvesicles, retrovirus-like vesicles, and apoptotic bodies. J. Neurooncol..

[16] Poon I.K.H., Parkes M.A.F., Jiang L., Atkin-Smith G.K., Tixeira R., Gregory C.D., Ozkocak D.C., Rutter S.F., Caruso S., Santavanond J.P. (2019). Moving beyond size and phosphatidylserine exposure: Evidence for a diversity of apoptotic cell-derived extracellular vesicles in vitro. J. Extracell. Vesicles.

[17] Coumans F.A.W., Brisson A.R., Buzas E.I., Dignat-George F., Drees E.E.E., El-Andaloussi S., Emanueli C., Gasecka A., Hendrix A., Hill A.F. (2017). Methodological Guidelines to Study Extracellular Vesicles. Circ. Res..

[18] Théry C., Witwer K.W., Aikawa E., Alcaraz M.J., Anderson J.D., Andriantsitohaina R., Antoniou A., Arab T., Archer F., Atkin-Smith G.K. (2018). Minimal information for studies of extracellular vesicles 2018 (MISEV2018): A position statement of the International Society for Extracellular Vesicles and update of the MISEV2014 guidelines. J. Extracell. Vesicles.

[19] Rossi C., Cicalini I., Cufaro M.C., Agnifili L., Mastropasqua L., Lanuti P., Marchisio M., De Laurenzi V., Del Boccio P., Pieragostino D. (2019). Multi-Omics Approach for Studying Tears in Treatment-Naïve Glaucoma Patients. Int. J. Mol. Sci..

[20] Falasca K., Lanuti P., Ucciferri C., Pieragostino D., Cufaro M.C., Bologna G., Federici L., Miscia S., Pontolillo M., Auricchio A. (2021). Circulating extracellular vesicles as new inflammation marker in HIV infection. AIDS.

[21] Brocco D., Lanuti P., Pieragostino D., Cufaro M.C., Simeone P., Bologna G., Di Marino P., De Tursi M., Grassadonia A., Irtelli L. (2021). Phenotypic and Proteomic Analysis Identifies Hallmarks of Blood Circulating Extracellular Vesicles in NSCLC Responders to Immune Checkpoint Inhibitors. Cancers.

[22] Pieragostino D., Cicalini I., Lanuti P., Ercolino E., di Ioia M., Zucchelli M., Zappacosta R., Miscia S., Marchisio M., Sacchetta P. (2018). Enhanced release of acid sphingomyelinase-enriched exosomes generates a lipidomics signature in CSF of Multiple Sclerosis patients. Sci. Rep..

[23] Brocco D., Lanuti P., Simeone P., Bologna G., Pieragostino D., Cufaro M.C., Graziano V., Peri M., Di Marino P., De Tursi M. (2019). Circulating Cancer Stem Cell-Derived Extracellular Vesicles as a Novel Biomarker for Clinical Outcome Evaluation. J. Oncol..

[24] Cela I., Cufaro M.C., Fucito M., Pieragostino D., Lanuti P., Sallese M., Del Boccio P., Di Matteo A., Allocati N., De Laurenzi V. (2022). Proteomic Investigation of the Role of Nucleostemin in Nucleophosmin-Mutated OCI-AML 3 Cell Line. Int. J. Mol. Sci..

[25] Pieragostino D., Lanuti P., Cicalini I., Cufaro M.C., Ciccocioppo F., Ronci M., Simeone P., Onofrj M., van der Pol E., Fontana A. (2019). Proteomics characterization of extracellular vesicles sorted by flow cytometry reveals a disease-specific molecular cross-talk from cerebrospinal fluid and tears in multiple sclerosis. J. Proteom..

[26] Buca D., D’Antonio F., Buca D., Di Sebastiano F., Simeone P., Di Girolamo R., Bologna G., Vespa S., Catitti G., Liberati M. (2022). Extracellular Vesicles in pregnancy: Their potential role as a liquid biopsy. J. Reprod. Immunol..

[27] Tkach M., Théry C. (2016). Communication by Extracellular Vesicles: Where We Are and Where We Need to Go. Cell.

[28] Tetta C., Ghigo E., Silengo L., Deregibus M.C., Camussi G. (2013). Extracellular vesicles as an emerging mechanism of cell-to-cell communication. Endocrine.

[29] Bittel D.C., Jaiswal J.K. (2019). Contribution of Extracellular Vesicles in Rebuilding Injured Muscles. Front. Physiol..

[30] Tidball J.G., Villalta S.A. (2010). Regulatory interactions between muscle and the immune system during muscle regeneration. Am. J. Physiol. Regul. Integr. Comp. Physiol..

[31] Tidball J.G. (2017). Regulation of muscle growth and regeneration by the immune system. Nat. Rev. Immunol..

[32] Rigamonti E., Zordan P., Sciorati C., Rovere-Querini P., Brunelli S. (2014). Macrophage plasticity in skeletal muscle repair. Biomed Res. Int..

[33] Wang Y., Zhao M., Liu S., Guo J., Lu Y., Cheng J., Liu J. (2020). Macrophage-derived extracellular vesicles: Diverse mediators of pathology and therapeutics in multiple diseases. Cell Death Dis..

[34] Antich-Rosselló M., Forteza-Genestra M.A., Monjo M., Ramis J.M. (2021). Platelet-Derived Extracellular Vesicles for Regenerative Medicine. Int. J. Mol. Sci..

[35] Vajen T., Mause S.F., Koenen R.R. (2015). Microvesicles from platelets: Novel drivers of vascular inflammation. Thromb. Haemost..

[36] Berckmans R.J., Lacroix R., Hau C.M., Sturk A., Nieuwland R. (2019). Extracellular vesicles and coagulation in blood from healthy humans revisited. J. Extracell. Vesicles.

[37] Melki I., Tessandier N., Zufferey A., Boilard E. (2017). Platelet microvesicles in health and disease. Platelets.

[38] Santilli F., Marchisio M., Lanuti P., Boccatonda A., Miscia S., Davì G. (2016). Microparticles as new markers of cardiovascular risk in diabetes and beyond. Thromb. Haemost..

[39] Gentile P., Garcovich S. (2020). Systematic Review-The Potential Implications of Different Platelet-Rich Plasma (PRP) Concentrations in Regenerative Medicine for Tissue Repair. Int. J. Mol. Sci..

[40] Marques L.F., Stessuk T., Camargo I.C.C., Sabeh Junior N., dos Santos L., Ribeiro-Paes J.T. (2015). Platelet-rich plasma (PRP): Methodological aspects and clinical applications. Platelets.

[41] Spakova T., Janockova J., Rosocha J. (2021). Characterization and Therapeutic Use of Extracellular Vesicles Derived from Platelets. Int. J. Mol. Sci..

[42] Sahu A., Clemens Z.J., Shinde S.N., Sivakumar S., Pius A., Bhatia A., Picciolini S., Carlomagno C., Gualerzi A., Bedoni M. (2021). Regulation of aged skeletal muscle regeneration by circulating extracellular vesicles. Nat. Aging.

[43] Wang H., Wang B. (2016). Extracellular vesicle microRNAs mediate skeletal muscle myogenesis and disease. Biomed. Rep..

[44] Nakamura Y., Miyaki S., Ishitobi H., Matsuyama S., Nakasa T., Kamei N., Akimoto T., Higashi Y., Ochi M. (2015). Mesenchymal-stem-cell-derived exosomes accelerate skeletal muscle regeneration. FEBS Lett..

[45] Zhu Y.-G., Feng X.-M., Abbott J., Fang X.-H., Hao Q., Monsel A., Qu J.-M., Matthay M.A., Lee J.W. (2014). Human mesenchymal stem cell microvesicles for treatment of *Escherichia coli* endotoxin-induced acute lung injury in mice. Stem Cells.

[46] Figliolini F., Ranghino A., Grange C., Cedrino M., Tapparo M., Cavallari C., Rossi A., Togliatto G., Femminò S., Gugliuzza M.V. (2020). Extracellular Vesicles From Adipose Stem Cells Prevent Muscle Damage and Inflammation in a Mouse Model of Hind Limb Ischemia: Role of Neuregulin-1. Arterioscler. Thromb. Vasc. Biol..

[47] Vajen T., Benedikter B.J., Heinzmann A.C.A., Vasina E.M., Henskens Y., Parsons M., Maguire P.B., Stassen F.R., Heemskerk J.W.M., Schurgers L.J. (2017). Platelet extracellular vesicles induce a pro-inflammatory smooth muscle cell phenotype. J. Extracell. Vesicles.

[48] Iyer S.R., Scheiber A.L., Yarowsky P., Henn R.F., Otsuru S., Lovering R.M. (2020). Exosomes Isolated From Platelet-Rich Plasma and Mesenchymal Stem Cells Promote Recovery of Function after Muscle Injury. Am. J. Sports Med..

[49] Brahmer A., Neuberger E., Esch-Heisser L., Haller N., Jorgensen M.M., Baek R., Möbius W., Simon P., Krämer-Albers E.-M. (2019). Platelets, endothelial cells and leukocytes contribute to the exercise-triggered release of extracellular vesicles into the circulation. J. Extracell. Vesicles.

[50] Ma Q., Bai J., Xu J., Dai H., Fan Q., Fei Z., Chu J., Yao C., Shi H., Zhou X. (2021). Reshaping the Inflammatory Environment in Rheumatoid Arthritis Joints by Targeting Delivery of Berberine with Platelet-Derived Extracellular Vesicles. Adv. NanoBiomed Res..

[51] Chandler W.L. (2013). Microparticle counts in platelet-rich and platelet-free plasma, effect of centrifugation and sample-processing protocols. Blood Coagul. Fibrinolysis.

[52] Ziemkiewicz N., Hilliard G., Pullen N.A., Garg K. (2021). The Role of Innate and Adaptive Immune Cells in Skeletal Muscle Regeneration. Int. J. Mol. Sci..

[53] Casolo A., Del Vecchio A., Balshaw T.G., Maeo S., Lanza M.B., Felici F., Folland J.P., Farina D. (2021). Behavior of motor units during submaximal isometric contractions in chronically strength-trained individuals. J. Appl. Physiol..

[54] Casolo A., Farina D., Falla D., Bazzucchi I., Felici F., Del Vecchio A. (2020). Strength Training Increases Conduction Velocity of High-Threshold Motor Units. Med. Sci. Sports Exerc..

[55] Marchisio M., Simeone P., Bologna G., Ercolino E., Pierdomenico L., Pieragostino D., Ventrella A., Antonini F., Del Zotto G., Vergara D. (2020). Flow Cytometry Analysis of Circulating Extracellular Vesicle Subtypes from Fresh Peripheral Blood Samples. Int. J. Mol. Sci..

[56] Brocco D., Simeone P., Buca D., Di Marino P., De Tursi M., Grassadonia A., De Lellis L., Martino M.T., Veschi S., Iezzi M. (2022). Blood Circulating CD133+ Extracellular Vesicles Predict Clinical Outcomes in Patients with Metastatic Colorectal Cancer. Cancers.

[57] Buca D., Bologna G., D’Amico A., Cugini S., Musca F., Febbo M., D’Arcangelo D., Buca D., Simeone P., Liberati M. (2020). Extracellular Vesicles in Feto-Maternal Crosstalk and Pregnancy Disorders. Int. J. Mol. Sci..

[58] Simeone P., Celia C., Bologna G., Ercolino E., Pierdomenico L., Cilurzo F., Grande R., Diomede F., Vespa S., Canonico B. (2020). Diameters and Fluorescence Calibration for Extracellular Vesicle Analyses by Flow Cytometry. Int. J. Mol. Sci..

[59] Lanuti P., Ciccocioppo F., Bonanni L., Marchisio M., Lachmann R., Tabet N., Pierdomenico L., Santavenere E., Catinella V., Iacone A. (2012). Amyloid-specific T-cells differentiate Alzheimer’s disease from Lewy body dementia. Neurobiol. Aging.

[60] Lanuti P., Simeone P., Rotta G., Almici C., Avvisati G., Azzaro R., Bologna G., Budillon A., Di Cerbo M., Di Gennaro E. (2018). A standardized flow cytometry network study for the assessment of circulating endothelial cell physiological ranges. Sci. Rep..

[61] Bansal H., Leon J., Pont J.L., Wilson D.A., Bansal A., Agarwal D., Preoteasa I. (2021). Platelet-rich plasma (PRP) in osteoarthritis (OA) knee: Correct dose critical for long term clinical efficacy. Sci. Rep..

[62] Potenza F., Cufaro M.C., Di Biase L., Panella V., Di Campli A., Ruggieri A.G., Dufrusine B., Restelli E., Pietrangelo L., Protasi F. (2021). Proteomic Analysis of Marinesco-Sjogren Syndrome Fibroblasts Indicates Pro-Survival Metabolic Adaptation to SIL1 Loss. Int. J. Mol. Sci..

