# Supplementary material for: Extracellular Vesicles in Regenerative Processes Associated with Muscle Injury Recovery of Professional Athletes Undergoing Sub Maximal Strength Rehabilitation
Source: Int J Mol Sci. 2022 Nov 29;23(23):14913. doi: 10.3390/ijms232314913 (PMC9739739; doi:10.3390/ijms232314913)
Supplement: Supplementary file 1 [file ijms-23-14913-s001.zip › ijms-2010086-supplementary.pdf]

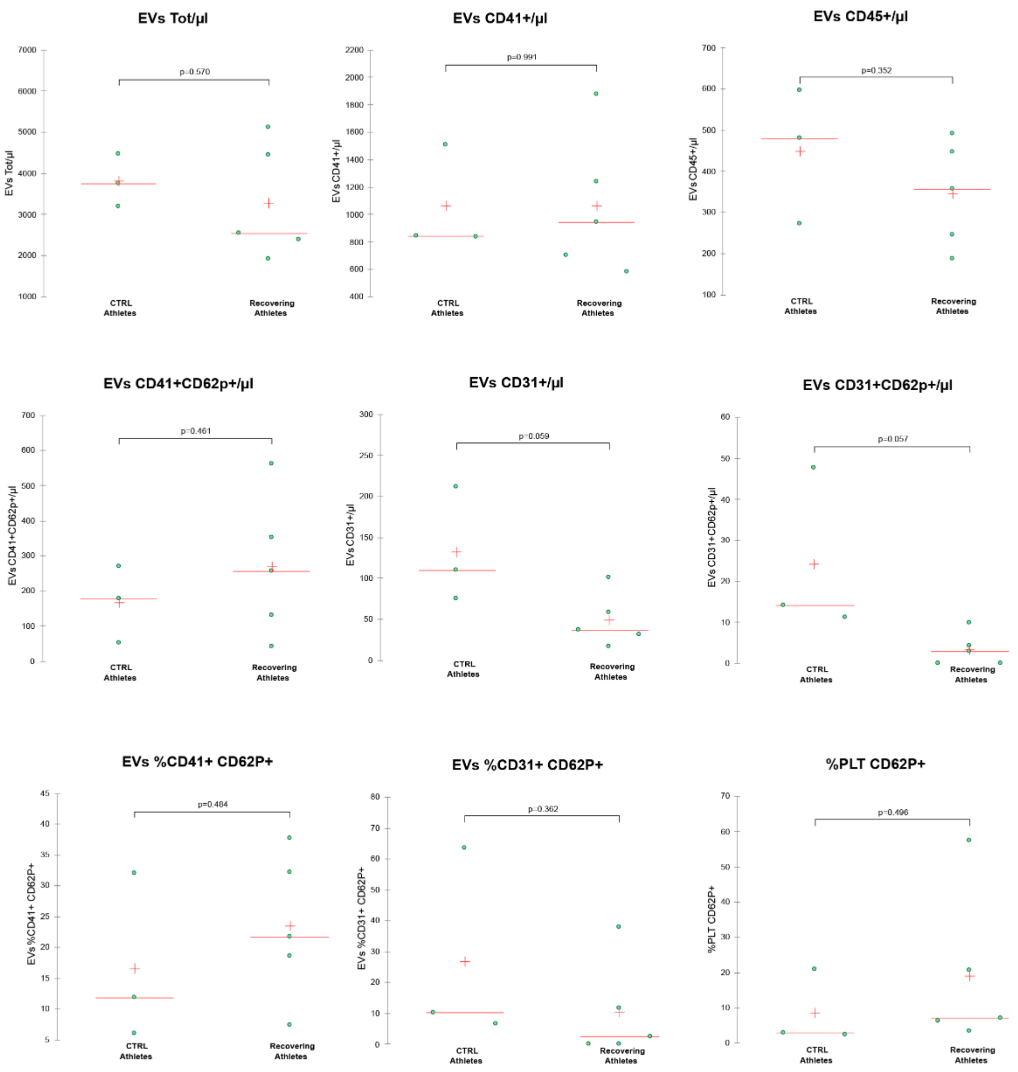

Figure S1. EVs counts from athletes recovering from muscle injuries compared to healthy volunteers . Different EV subtypes were identified and counted in athletes recovering from muscle injuries (T0, N=5) and healthy volunteers (N=3). Green dots represent single analyzed subjects, the red horizontal line shows the median of the cohort values, while the cross represents the mean of the values.

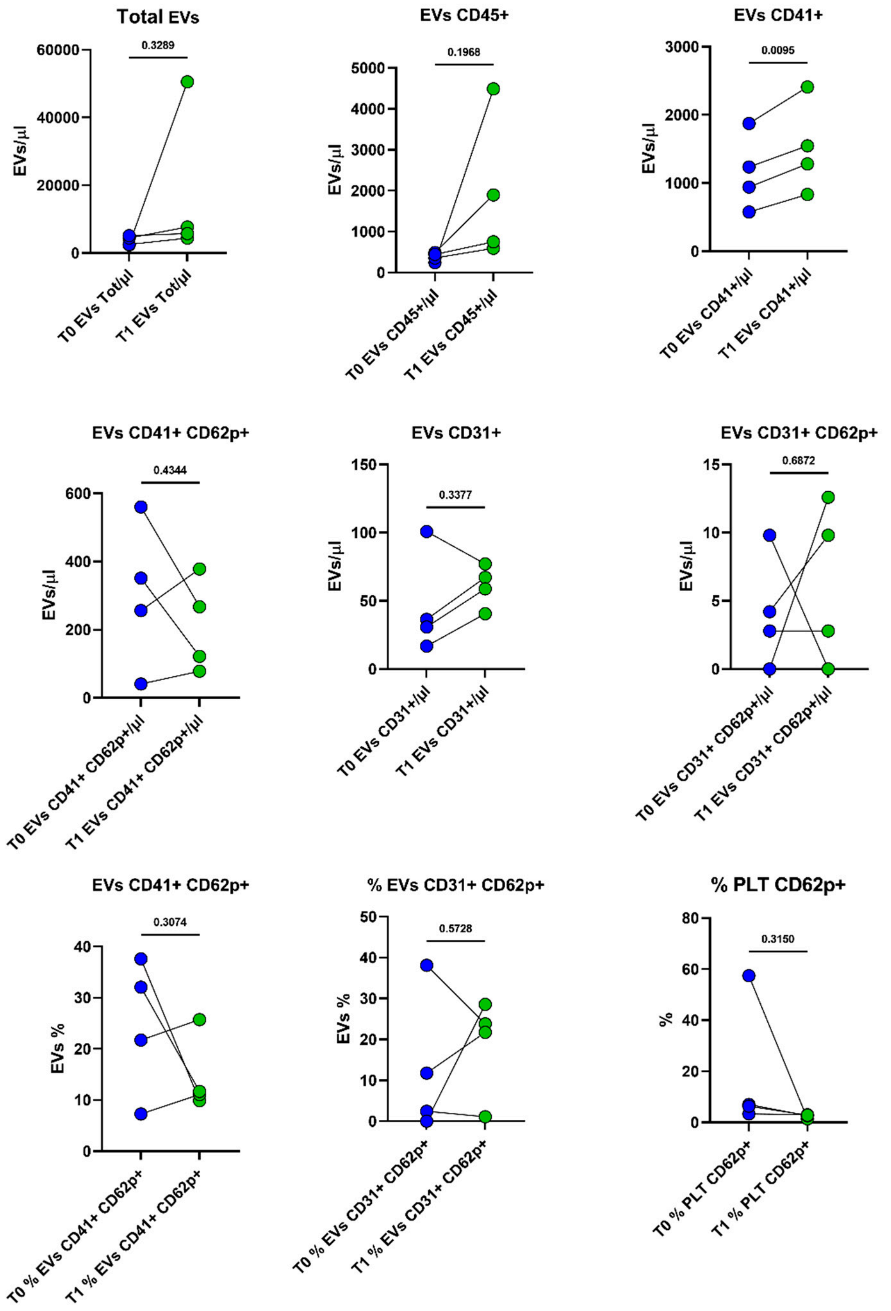

Figure S2. EVs circulating in the peripheral blood of athletes recovering from muscle injuries. Graphs showing absolute counts and percentages of different EV circulating subtypes, analyzed before (T0, blue dots) and after (T1, green dots) the rehabilitation program.

Table S1. Reagent Mix

Specificity	Clone	Fluorochrome/Channel	Amount per test	Catalogue Number
Phalloidin	-	FITC	0.5ul	626267 (Custom Kit)
LCD	-	APC	0.5ul	626267 (Custom Kit)
CD41a	HIP8	PE	2.5ul	555467
CD31	WM59	PE-Cy7	0.5ul	563651
CD62P	AK-4	BV421	3ul	564038
CD45	HI30	BV510	1ul	563204

Fluorescein isothiocyanate (FITC); Allophycocyanin (APC); Phycoerythrin (PE); PE-Cyanine 7 (PE-Cy7), BV=Brilliant Violet.
